# Novel Influenza A (H1N1/09) Virus Infection during Pregnancy in a Kidney Transplant Recipient

**DOI:** 10.1155/2011/636030

**Published:** 2011-07-09

**Authors:** Lars Rothermund, Reinhold Kreutz, Frieder Keller

**Affiliations:** ^1^Institut für Klinische Pharmakologie und Toxikologie, Charité Universitätsmedizin Berlin, Charitéplatz 1, 10117 Berlin, Germany; ^2^Kuratorium für Heimdialyse und Nierentransplantation Nierenzentrum Ulm, Erlenstrasse 40, 89081 Ulm, Germany; ^3^Sektion Nephrologie, Abteilung Innere Medizin I, Universitätsklinikum, Albert-Einstein Allee 23, 89070 Ulm, Germany

## Abstract

We report the first case of a pregnant renal transplant patient with H1N1/09 infection. The woman showed a mild clinical course after diagnosis of H1N1/09 infection and therapy with oseltamivir (2 × 45 mg per day). After delivery by cesarean section, the neonate exhibited moderate respiratory and circulatory dysregulation, which spontaneously normalised a few days *postpartum*. In conclusion, rapid diagnosis of H1N1/09 and dose-adapted therapy with oseltamivir resulted in successful delivery of a healthy infant in our renal transplanted patient but emphasized the need for consequent vaccination strategies in pregnant transplant recipients for new influenza A pandemics in the future.

## 1. Introduction

The influenza A H1N1/09 pandemic virus was especially threatening for pregnant women [1] and patients on immunosuppressive therapies, as they are at increased risk for complicated and prolonged infection and mortality [1–3]. 

In this paper, we present the first case of H1N1/09 virus infection during pregnancy after kidney transplantation and describe clinical presentation, diagnosis, therapy, and outcome, specifically addressing treatment with oseltamivir.

## 2. Case Presentation

A woman aged 28 years at 18 weeks of gestation (gravida 3, para 0) with a 2-day history of myalgias, cephalgia, fatigue, productive cough, sore throat, and subfebrile temperature (38.2°C) consulted her nephrologist in an outclinic setting. 

The patient reported a complex previous history of autosomal-recessive juvenile nephronopthisis type 1 and treatment with peritoneal dialysis (for 2 years). The patient had received two kidney transplant grafts, the first at the age of 15 (deceased donor kidney), which was lost due to chronic transplant failure. At the age of 19, she received a live donation from her father with a stable kidney transplant function since then. 

13 months before the present event, she terminated oral contraception, while the immunosuppressive regimen was switched from tacrolimus/mycophenolate mofetil to prednisolone (1 × 5 mg per day)/ cyclosporine (2 × 110 mg per day), and antihypertensive medication was changed to alpha–methyldopa in preparation for desired pregnancy.

Two spontaneous abortions had occurred (at 4 and 6 weeks of gestation, resp.) without major complications before stable pregnancy was achieved. 

With current presentation, renal transplant function was unchanged compared to previous visits (serum creatinine concentration 1.1 mg/dL, eGFR (MDRD) 57 mL/min/1.73 m^2^). In contrast, serum C-reactive protein concentration (CrP) was elevated 11-fold (33 mg/L) with normal leucocytes (6.5 G/L) and a slightly elevated monocyte count (13%). Cytomegalovirus quantitative polymerase chain reaction (PCR) and Epstein Barr virus serology were negative. 

2 nasal and 1 pharyngeal specimens were collected; all 3 of them were positive by real-time reverse transcription-PCR (rRT-PCR) for H1N1/09 virus. The patient reported to have no vaccination for seasonal influenza A or H1N1/09.

Treatment with oseltamivir was started at an adapted dose of 2 × 45 mg per day, in addition to treatment with the antibiotic amoxicillin 2 × 750 mg per day.

This treatment resulted in prompt clinical improvement after 2 days and was continued until CrP concentration normalized after 5 days. Renal transplant function (serum creatinine 1.1-1.2 mg/dL) and cyclosporine trough levels (82–113 *μ*g/L) remained stable during treatment. 

Obstetric ultrasound examination 3 weeks after onset of H1N1/09 infection did not reveal any alterations in fetal status. In the following weeks pregnancy and fetal development were uncomplicated. 

At 37 + 2 weeks of gestation delivery was performed by primary cesarean section with intubation narcosis. The newborn (body weight 3300 g, APGAR score 8/10/10) had respiratory arrhythmias in the first hours *postpartum* together with decreases of oxygen saturation (SpO_2_). During postpartal day 3 and 4 again several decreases of SpO_2_ and bradycardias were observed. Event recording revealed signs of immaturity of the respiration centre with apnoeas. Continued monitoring demonstrated a complete respiratory stabilisation after postpartal day 8. The mother recovered without problems, and renal graft function remained stable in the postpartal period. Thus, hospital discharge of the mother and the healthy infant was possible on day 9 after delivery. The newborn developed without further complications in the following 3 months.

## 3. Discussion

In this paper we describe the first case of H1N1/09 virus infection in a pregnant woman with a kidney transplant. We followed general treatment recommendations of national and international guidelines [2, 3].

With reduced renal function oseltamivir has to be dose adapted [4]. Although estimation of GFR by the MDRD formula may underestimate GFR during pregnancy [5], we relied on eGFR values since rapid determination by clearance measurements was not feasible in our pregnant patient, and a deterioration of kidney transplant function was a possible complication due to systemic infection. Because eGFR was <60 mL/min/1.73 m^2^, we reduced the standard dose of 150 mg to 90 mg oseltamivir per day (2 × 45 mg). Oseltamivir is not substrate for, or inhibitor of, cytochrome P450 isoenzymes [4], which explains the stable cyclosporine levels in our patient. Moreover, we did not reduce immunosuppressive therapy due to H1N1/09 viral infection, because infectious symptoms were controlled immediately after onset of oseltamivir therapy. For the same reasons, and due to possible unknown effects of oseltamivir in human pregnancy, we performed only a 5-day treatment course, as approved at the time of onset of H1N1/09 infection in our patient [2, 3].

With regard to the initial respiratory and circulatory instability of the neonate, we cannot exclude consequences of the H1N1/09 infection or the treatment with oseltamivir as possible causes. The effects of influenza during pregnancy, whether mediated directly by the virus or by fever or other events secondary to the underlying infection, are not yet well understood [1].

Moreover, transplacental transfer of oseltamivir was very limited and not detectable at normal therapeutic doses in an *ex vivo* human placenta model [6].

Taken together, we cannot define the underlying cause of the postpartal complications of the neonate, since the findings could also be related to effects of general anesthesia, delivery by caesarean section, underlying impairment of the renal graft, and/or immunosuppressive therapy [7] rather than to H1N1/09 infection or oseltamivir treatment.

## 4. Conclusion

In our pregnant patient with a kidney transplant graft, rapid diagnosis of H1N1/09 infection and dose-adapted therapy with oseltamivir already 2 days after onset of symptoms, together with continuation of immunosuppressive regimen, resulted in a successful and well-tolerated treatment of H1N1/09 infection and delivery of a healthy neonate. Postpartal complications due to H1N1/09 infection during pregnancy and oseltamivir treatment cannot be ruled out. 

Therefore, this paper unfolds and emphasizes that pregnant women with a renal transplant graft should be counselled about the importance of vaccination in order to avoid possible complications of H1N1/09 infection or antiviral treatment. Valid vaccination guidelines for the influenza A pandemics we will face in the future have to be developed, and accurate information regarding the safety of antiviral agents in pregnant women and patients on immunosuppressive therapies are needed.
